# Signing data citations enables data verification and citation persistence

**DOI:** 10.1038/s41597-023-02230-y

**Published:** 2023-06-27

**Authors:** Michael J. Elliott, Jorrit H. Poelen, José A. B. Fortes

**Affiliations:** 1 University of Florida, Gainesville, FL USA; 2 Ronin Institute, Montclair, NJ USA; 3 UC Santa Barbara Cheadle Center for Biodiversity and Ecological Restoration, Santa Barbara, CA USA

**Keywords:** Research data, Biodiversity

## Abstract

Commonly used data citation practices rely on unverifiable retrieval methods which are susceptible to content drift, which occurs when the data associated with an identifier have been allowed to change. Based on our earlier work on reliable dataset identifiers, we propose signed citations, i.e., customary data citations extended to also include a standards-based, verifiable, unique, and fixed-length digital content signature. We show that content signatures enable independent verification of the cited content and can improve the persistence of the citation. Because content signatures are location- and storage-medium-agnostic, cited data can be copied to new locations to ensure their persistence across current and future storage media and data networks. As a result, content signatures can be leveraged to help scalably store, locate, access, and independently verify content across new and existing data infrastructures. Content signatures can also be embedded inside content to create robust, distributed knowledge graphs that can be cited using a single signed citation. We describe applications of signed citations to solve real-world data collection, identification, and citation challenges.

## Introduction

### Rise of the Internet and unreliable content references

Initially funded by DARPA and the National Science Foundation, the Internet first emerged as a network of interconnected academic computer sites in the early 1980s. Subsequent introduction of domain name services (e.g., Domain Name System (DNS)), standard transfer protocols (e.g., TCP/IP, HTTP), and the HyperText Markup Language (HTML) led to the global adoption of the Internet as the dominant digital-content networking infrastructure in the first decade of the 21st century^1^. However, evidence soon appeared that Internet-accessible content associated with URLs often changed (content drift) or became unavailable (link rot). Despite the realization that URLs were unsuitable for reliably referencing digital data, these brittle resource locators became common practice in citing digital data in academic literature^2^. In other words, content was being cited by its observed location (e.g., a temporary Internet address) rather than its assigned identity or the information it contained (e.g., unique content characteristics).

Concerted efforts have since been made to prevent link rot and content drift for digital content, especially those that have importance in research. Centralized data repositories (e.g., Internet Archive (https://archive.org), Zenodo (https://zenodo.org), Dryad (https://datadryad.org), Dataverse (https://dataverse.org); a more comprehensive list is available at the Registry of Research Data Repositories (https://re3data.org)) combat link rot and content drift by adhering to good data management practices, such as versioning, reliable storage systems, and designated permalinks for locating data. Furthermore, Persistent Identifiers (PIDs, e.g., ARKs^3^ and DOIs^4^) reduce the effects of link rot by decoupling content identification from content location, such that data may be moved without necessarily losing identifier resolvability. Metadata for persistent identifiers (e.g., mappings between DOIs and URLs) are kept up-to-date through combinations of auditing mechanisms and adaptive stewardship policies. Even when data become inaccessible, persistent identifiers allow continued referencing of the data and continued access to descriptive metadata. Meanwhile, socio-economic incentives provide strong support for the continued maintenance of persistent identifiers such as DOIs.

Recent years have seen a trend toward publishing and citing digital scientific data. Unlike most published texts, datasets in some research domains tend to evolve over time through ongoing curation and data collection efforts^5^. For such datasets, specific versions may need to be cited to keep experimental results reproducible. However, modern citation methods lack verification mechanisms for confirming retrieval of the correct version of cited digital content. This can be problematic, as persistent identifiers are not always used to cite specific versions of content, but rather are often assigned to evolving digital objects. Without verifiability, even persistent identifiers that are assigned to a single content version can be affected by undetected content drift, whether due to administration errors or random data corruption.

A simple approach to digital content verification is to examine the bits that make up the content and count the number of 1s (or 0s), then record the count’s parity – whether it is even or odd. The parity can be encoded as a parity bit, where the bit is set to 1 when the count is even, or 0 when it is odd. Then, changes in the content can be detected by recomputing the parity bit and comparing it to the previously computed value. If the bits differ, the content must be different from the original. If the bits do match, the content might be the same as the original, but it is not guaranteed; if an even number of bits were different, the parity of the resulting content would not change, leading to a false positive verification. More sophisticated verification algorithms can achieve much lower rates of false positives. Specifically, some cryptographic hashing algorithms (e.g., SHA-256^6^) produce fixed-size content encodings – called content hashes – for which the probability of a false positive is practically zero^7^. For example, if the SHA-256 hashing algorithm produces the same hash for two pieces of content, it is safe to assume that they are identical. Likewise, if the hashes are different, the contents must be different. Like parity bits, content hashes are derived from content, and therefore can be reliably recomputed by anyone in possession of the content. We refer to an identifier that contains a verifiable content hash as a content signature.

We propose extending data citation practices to allow the inclusion of standards-based, fixed length, verifiable, unique content signatures (e.g., a SHA-256 content-hash URI) in customary scientific citations. We refer to a citation that includes such a content signature as a signed citation. By including content signatures that are unique and verifiable, signed citations are robust, as they always reference the originally cited data even when located copies of the data are modified (content drift) or become inaccessible (link rot). Furthermore, signed citations can enable readers to discover new locations of cited data using public content signature registries, if available^5^. If content is embedded with signed citations for other content, entire data networks can be referenced, verified, and potentially retrieved using a single signed citation.

## Related Work

The use of unique content hashes is a core principle in many decentralized data-oriented applications, such as version control systems (e.g., Git), content-addressed storage (e.g., EMC Centera^8^), content delivery networks (e.g., Akamai^9^, CloudFlare CDN), information-centric networking (e.g., DONA^10^, NetInf^11^), peer-to-peer networking (e.g., Secure Scuttlebutt^12^, BitTorrent), and blockchains (e.g., Bitcoin^13^). Purely content-based identifiers – free of external information requirements imposed by mechanisms such as cryptographic access control^14^ and hash salting^15^ – may be reused and recalculated by independent parties, allowing the identifiers to outlive and outgrow the original systems they were designed for. This property has enabled and driven the continued development of interoperable yet independent software and information systems without compromising old identifiers. Such systems are regularly leveraged by researchers to increase the longevity and accessibility of published experimental methods and results^16–18^. Version control systems such as Git are convenient for preserving experimental methods and datasets with meticulous provenance logging and content identification, made accessible through online platforms like GitHub (https://github.com) and Zenodo (https://zenodo.org). Some data warehouses, such as Zenodo and Software Heritage^19^ (https://www.softwareheritage.org), allow their collected data to be retrieved by past locations or content-based identifiers even after becoming unavailable at their original locations. This is one of the primary objectives of the Software Heritage archive – to preserve access to versioned copies of open-source projects on online platforms such as GitHub and CRAN long after they become inaccessible at their original URL locations^19^. Furthermore, decentralized public ledger techniques such as blockchain have proven to be successful at sustaining practically uncompromisable provenance chains between digital objects through the use of content-based identification principles^20^. However, despite the availability of infrastructure for identifying, locating, and describing data using content hashes, their use in current citation practices lacks the standardization and well-known resolution mechanisms required for more widespread adoption.

Although current scientific data citation practices predominantly rely on persistent identifiers^21^ (PIDs) such as Digital Object Identifiers^4^ (DOIs), Archival Resource Keys^3^ (ARKs), and Life Sciences Identifiers (LSIDs, https://omg.org/spec/LIS), several hash-based identifier schemes – such as Universal Numerical Fingerprints (UNF)^22^, Trusty URIs^23^, named identifiers^24^ (ni), magnet links (https://magnet-uri.sourceforge.net/), content-hash URIs (https://github.com/hash-uri/hash-uri), and Software Heritage Persistent Identifiers^25^ (SWH-IDs) – have been proposed for inclusion in data citations^26^. Some of these identifier schemes impose constraints on the types of data that can be identified. For example, UNFs are well-suited for citing tabular datasets by forming a hash of the information they contain, rather than their exact digital content, such that the same identifier may be calculated for non-identical datasets when their differences are non-semantic in nature (for example, differences in spaces, record order, numerical precision, or digital format). Consequently, UNFs cannot be used to cite other types of data, such as images, videos, audio, or any type of binary data that is not representable numerically or textually, including data archives that contain such data elements. Similarly, Trusty URIs of type R can only be computed for Resource Description Framework (RDF) data and ignore various non-semantic bit-level differences^23^. While data-type specific identifier schemes such as UNFs and Trusty URIs are useful in specific contexts, a more general approach (e.g., Named Identifiers, magnet links, or content-hash URIs) is required for the hash-based citation of data types that lack a tailored identifier scheme. Additionally, UNFs, TrustyURIs, and SWH-IDs assume the use of specific hashing algorithms. In our Discussion section, we describe benefits of being able to distinguish between different digital representations of the same or similar data, as well as advantages of using schemes with hashing algorithm flexibility.

Content signatures can be used to build and uniquely identify complex, distributed data networks^13,23^. For example, if a dataset contains the content signatures of other datasets, a logical link is formed between the dataset and those that it references. By repeating this process, citation graphs of any size can be constructed, then cited using a single fixed-length content signature. Trusty URIs use this feature of content signatures to build and identify provenance graphs for publications, encoded in RDF, which conveys structured descriptions of the properties of, and relationships between, uniquely identified resources. Then, Trusty URIs that are resolvable (e.g., embedded in URLs) can be followed to discover the origins of a publication^23^. Such provenance graphs are robust because the content signatures used to link content can only ever identify those exact contents, such that the no content referenced in the graph (including the RDF that forms the graph) can be altered (to the extent that the identifier distinguishes between objects, e.g., adding or removing whitespace does not alter a dataset’s Trusty URI) without changing the hash of the entire graph. This property is also fundamental to the design of blockchains, most notably used by cryptocurrencies such as Bitcoin to create and evolve robust transaction ledgers^13^, and can be leveraged in scientific citations to allow the origins of scientific findings to be more reliably traced as well as allow the authors of published datasets to be more readily credited for their contributions.

### Contributions and origin of work

Evidence for the reliability of content signatures as references has been described in^5^, where they were used to detect and quantify link rot and content drift over time for biodiversity dataset URLs registered with several data aggregators. We found that 20% to 75% of dataset URLs exhibited link rot or content drift over a span of two years. Specifically, link rot was detected in 5% to 70% of URLs, and content drift in 0.05% to 66%. Although we did not investigate the frequencies of link rot and content drift for persistent identifiers such as DOIs, we realized that such identifiers did not generally provide any means of verifying the identity of associated data, whereas content signatures could. In this paper, we build upon our earlier work^5^ by formalizing the role of content hashes in content signatures used to construct signed citations and documenting real-world usages of content signatures to create repeatable, reproducible workflows and robust provenance graphs. We discuss how content signatures enable verifiable content retrieval and discovery using decentralized content registries, repositories, and search indexes, with the potential for leveraging existing infrastructures.

## Methods

### Signed citations are robust

A content signature is a content identifier that contains the following two components: a cryptographic hash of the identified content, and a description of which cryptographic hashing algorithm was used to compute the hash. A signed citation is simply a data citation that includes a unique and verifiable content signature of the cited data. To create a signed citation, the following two criteria need to be fulfilled:

1. the referenced content is digital
2. means are available to calculate a content hash of that digital content (e.g., via cryptographic hashing algorithms such as SHA-256^6^)

A signed citation can then be formed by including all the elements of a traditional citation (e.g., author, title, publisher, and publication date) as well as the content signature for the cited data. In example 1, below, we illustrate the construction of a signed citation for an image of a bee specimen.

**Fig. 1:**
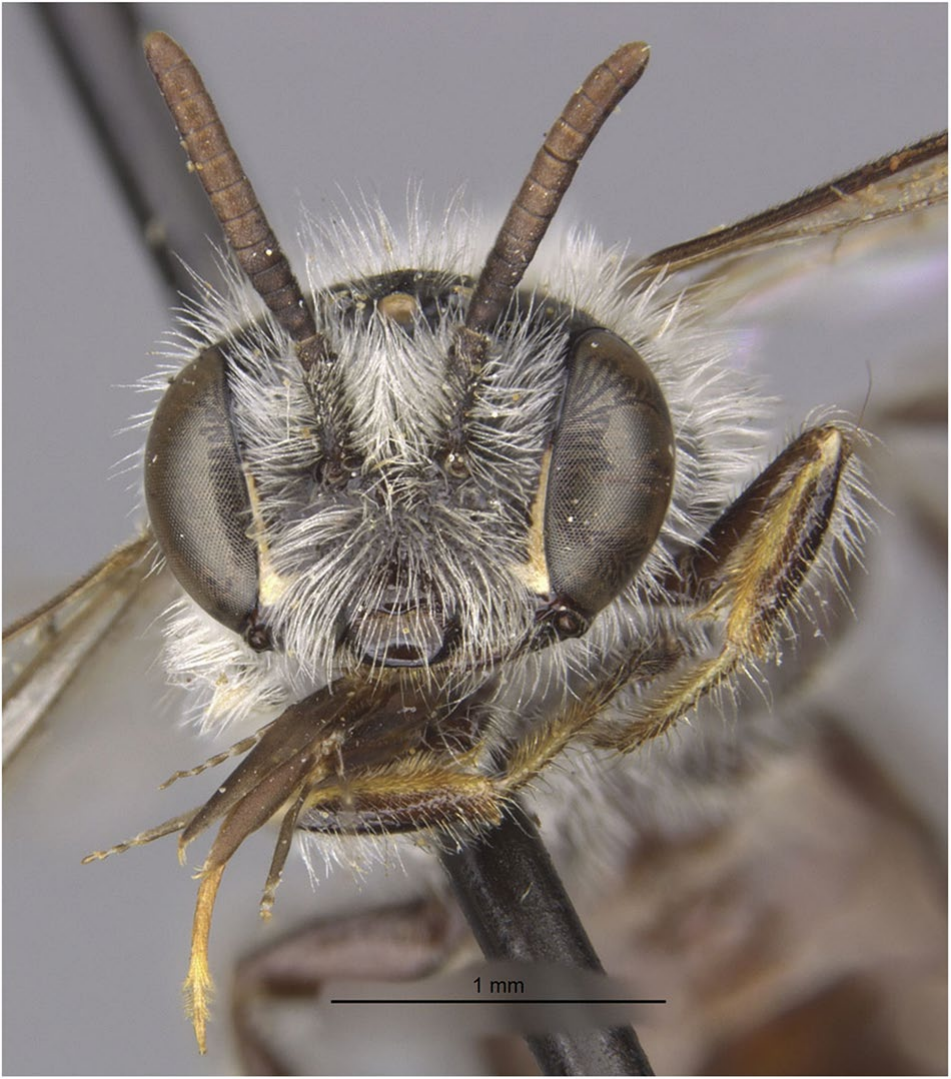
Headshot of *Nomadopsis puellae* (Cockerell, 1933) specimen MCZ:Ent:17219, used with permission from the Museum of Comparative Zoology, Harvard University, ©President and Fellows of Harvard CC-BY-NC-SA 4.0. The image was not modified from its original form. The image has the SHA-256 content signature hash://sha256/edde5b2b45961e356f27b81a3aa51584de4761ad9fa678c4b9fa3230808ea356. A signed citation for this image is provided in example 1.

*Example 1*. In this example, we verifiably cite a digital image of the bee specimen MCZ:Ent:17219 held at the Museum of Comparative Zoology, Harvard University. A rendering of the image is shown in Fig. 1, below.

Because the image is digital, its content hash can be computed using a cryptographic hashing algorithm. We chose to use the SHA-256 algorithm to compute a hash of the image and formed the following content signature:


hash://sha256/edde5b2b45961e356f27b81a3aa51584de4761ad9fa678c4b9fa3230808ea356


which can be listed alongside the author, date, title, and location the content was retrieved from to form the following signed citation:

Museum of Comparative Zoology, Harvard University. 2021. Head Frontal View of MCZ:ENT:17219 Nomadopsis puellae (Cockerell, 1933) hash://sha256/edde5b2b45961e356f27b81a3aa51584de4761ad9fa678c4b9fa3230808ea356 Accessed at http://mczbase.mcz.harvard.edu/specimen_images/entomology/large/MCZ-ENT00017219_Spinoliella_puellae_hef.jpg on 2021-12-07.

A future reader who finds the cited content may verify its identity by recomputing the content hash listed in the signed citation.

The signed citation in example 1 begins as a traditional citation, attributing the source of the image and its publication date, along with descriptive text. We then supplement this information with a content signature of the image. For convenience, we also cite the Internet location from which we retrieved the image, as well as the date of retrieval. Note that hash://sha256/edde5b2b45961e356f27b81a3aa51584de4761ad9fa678c4b9fa3230808ea356 is the content signature of the image, expressed as a content-hash URI, which has the general form of hash://[hash function]/[hash value] (e.g., hash://sha256/abc…). The notation provides an intuitive way to indicate that the hexadecimal sequence (where each character can be a digit or one of the letters in the set {a, b, c, d, e, f}) is a content hash and which hash function was used to generate the hash (e.g., sha256 indicates the SHA-256 algorithm). The hash function takes binary content as input and computes a content hash. In the above content signature, the binary content is the digital image from example 1, and the SHA-256 algorithm was used to generate the 256-bit hash that is encoded in the content signature as a 64-character hexadecimal sequence (each character representing 4 bits).

Hash functions are deterministic; given a specific digital content as input, a hash function will always produce the same hash, regardless of when, where, or by whom the hash is calculated. This means that the association between a hash and digital content can be verified by re-running the hash function to reproduce the hash. Furthermore, secure hash functions such as SHA-256 provide statistical guarantees that no two inputs will be assigned the same hash^6^. In other words, if a hash function produces different hashes for two pieces of digital content, their contents must be non-identical. Therefore, a hash is uniquely associated with exactly one digital content. By using content signatures that specify both a hash and a hash function, the association between a content signature and digital content is both unique and verifiable.

By including content signatures that are unique, verifiable, and location-agnostic, signed citations are resistant to both link rot and content drift in the sense that both can be detected and potentially repaired. If locations (e.g., URLs) listed in a signed citation become inaccessible, the reader may consult a content signature registry (if one is available) to look up alternative locations of the content identified by the content signature. If content is retrieved, content drift can be detected by recomputing its hash and checking whether it differs from the cited hash, in which case the reader can attempt to find an alternative location. Because the uniqueness and persistence^27^ of content signatures as identifiers cannot be corrupted by link rot and content drift, we say that signed citations are robust.

### Recursive signed citations form robust citation graphs

Now that we’ve established and exemplified a standards-based (e.g., using SHA-256^6^, URI^28^, and hexadecimal notation^29^) way to verifiably cite a single digital image, we will show by example how to cite a collection of digital data containing multiple images and associated metadata.

*Example 2*. A basic form of a collection description may look like a reference list:

Museum of Comparative Zoology, Harvard University. 2021. Head Frontal View of MCZ:ENT:17219 Nomadopsis puellae (Cockerell, 1933) hash://sha256/edde5b2b45961e356f27b81a3aa51584de4761ad9fa678c4b9fa3230808ea356 Accessed at http://mczbase.mcz.harvard.edu/specimen_images/entomology/large/MCZ-ENT00017219_Spinoliella_puellae_hef.jpg on 2021-12-07.

Museum of Comparative Zoology, Harvard University. 2021. Habitus Lateral View of MCZ:ENT:17219 Nomadopsis puellae (Cockerell, 1933) hash://sha256/8d49bd24f6ba300b4de44fd218b53294f4cc0106cd9631018ef819b38345c75d Accessed at http://mczbase.mcz.harvard.edu/specimen_images/entomology/large/MCZ-ENT00017219_Spinoliella_puellae_hal.jpg on 2021-12-07.

A signed citation can be generated for the entire collection description by computing the SHA-256 hash of an ASCII encoding^30^ of the text, then including a content signature (in this case, a content-hash URI) that specifies the hash and the hashing algorithm:

Some author, 2021. A collection of signed citations for various images. Hash://sha256/fe21dbf7e3ac1f9f82afa303a927015ada16ff84571e1fe21914c7053f00fb59 Accessed at https://example.org/citations.txt on 2021-12-08.

Because the collection description in example 2 robustly cites the two images using signed citations, the signed citation of the collection description also robustly cites the images. After retrieving the cited collection description and verifying its content signature, correct retrieval of the cited images can be similarly verified using the content signatures listed in the collection description. This demonstrates that signed citations can recursively cite other signed citations to form robust, traversable citation graphs. The process demonstrated in the example can be used to robustly cite any number of contents using a single signed citation.

### Robust citation graphs can be annotated to form evolving knowledge graphs

**Fig. 2:**
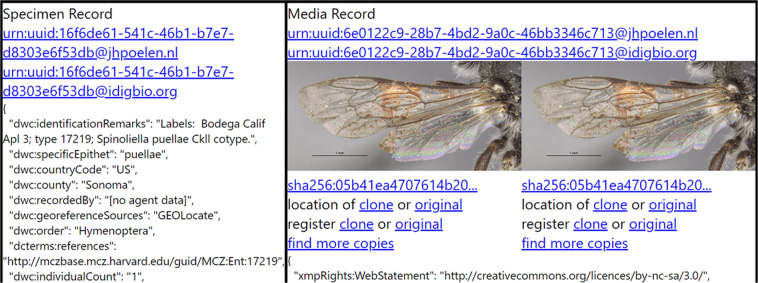
A screenshot of a machine-generated interactive web page visualizing the collection description cited in example 3. The web page displays 10 specimen records and their 39 associated media records, as well as information about where the data came from, their content signatures, and where they can be accessed. The web page was accessed at https://jhpoelen.nl/bees. The depicted photograph is included with permission from the Museum of Comparative Zoology, Harvard University, ©President and Fellows of Harvard CC-BY-NC-SA 4.0. The image was not modified from its original form.

A more elaborate version of the digital collection description in example 2 can be considered. Citation graphs formed by recursive signed citations can be annotated with descriptions of cited objects and their semantic relationships with other cited objects to form robust knowledge graphs. Example 3, below, cites a collection description that includes a semantic representation of the provenance (or origination) of bee images using the PROV Ontology^31^. This representation includes a description of the image origins and the software tools used to discover and collect them, namely a tool called Preston^32^ and the iDigBio Web Application Programming Interface (i.e., iDigBio’s Web API, https://search.idigbio.org). The collection description cited in example 3 includes the same references as the one in example 2, but expresses the observed origins (e.g., the discovery process, including location, date of observation, and intermediate data) of the images in the machine-readable Resource Definition Framework^33^ (RDF). Whereas example 2 is geared toward human readers, example 3 is easy for machines to read so that, for example, it may be used to re-generate example 2 using programmatic text manipulation or RDF query languages such as SPARQL^34^, or even generate an interactive web page (Fig. 2) containing renderings of the images and descriptions of how they were discovered.

*Example 3*. The following is a signed citation of a machine-readable collection of 39 digital bee images^35^:

A biodiversity dataset graph: https://jhpoelen.nl/bees. 2020. hash://sha256/85138e506a29fb73099fb050372d8a379794ab57fe4bfdf141743db0de2b985c

The collection uses PROV Ontology to describe how and from where the images were collected. This information is expressed using machine-readable RDF triples (i.e., a list of statements, each expressing a relationship between two objects) encoded as N-Quads^33^. A sample from the collection text is provided below.

<https://search.idigbio.org/v2/view/mediarecords/9eb732e4-ee0a-4114-b99f…

<hash://sha256/391c3a89b1f654b7f5f375ea14bc5b401815486539363d4641579bbd5…

<e5622c28-3584-4f2e-a2e1-4c331592ac5e http://www.w3.org/ns/prov#generated… [1637 more lines]

Text-processing tools were used to transform the encoded RDF statements from the cited collection into the more human-friendly interactive web page depicted in Fig. 2, below.

Figure 2 shows that the images described in media records on the right side are associated with specimen records on the left. These associations are recorded in RDF statements that form robust bidirectional links between media and specimen records using their content signatures. Furthermore, the links described in the cited collection form a knowledge graph that can be analyzed – and even aggregated with other graphs – to infer more complex relationships between content, such as finding that different specimen records share an associated image.

The collection description in example 3 can be extended and annotated by creating and citing a new collection description that references the former alongside new information. In other words, signed citations provide a formal and extensible way to assign context and meaning to digital content. This allows for recursive encapsulation (a reference to other references) and enrichment of the metadata, and thus offers a general, extensible, and robust approach to describing digital resources and how they came about. In our supplementary document, we provide real-world examples of evolving graphs where each version of the graph is an extension of the previous version.

While example 3 cites a relatively modest collection containing only 39 images (including the 2 images referenced in example 2) related to 10 specimen records, the generation of content signatures for much larger collections (e.g.,^36^) is possible and efficient using commodity computing resources.

## Results: real world applications

In our Methods section, we showed how signed citations can be used to robustly cite digital resources (example 1), to form citation graphs of digital resources (example 2), and to form citable knowledge graphs that describe links across heterogeneous digital resources and associated metadata (example 3). We have applied these capabilities to several real-world data organization challenges, including:

- Compiling and citing distributed collections of digitized images hosted at different locations – In our supplementary document, we describe how we are applying the recursive citation techniques from example 2 to compile a manifest of unique images and their locations in the developing Big-Bee image collection^37^, annotated with metadata linking the images to GBIF specimen records using techniques described in example 3.
- Discovery and versioning of datasets registered with biodiversity data aggregators over time – In our supplementary document, we describe how we have been able to collect hundreds of thousands of versioned biodiversity datasets registered with iDigBio, GBIF, and BioCASe. Using the recursive citation techniques in example 2, we are able to robustly cite all of the collected datasets using a single signed citation. Furthermore, our collection process is recorded in meticulous provenance logs that can be indexed and queried to select datasets of interest across all three aggregators.
- Stabilizing location-based references used by existing software – We used signed citations to mitigate the effects of link rot and content drift in Internet URLs used as dataset identifiers^38^. By mapping URLs to the content signatures of the contents observed at those locations^39^, URLs can be used as aliases for content signatures, where the content signatures are resolved to retrieve content instead of re-querying the URLs. This is discussed at greater length in our supplementary document.

## Discussion

### Extending customary citation practices

Increasingly, digital datasets are being published with assigned identifiers, then cited in papers as the basis for repeatable experiments. To help future readers find and verify data, customary citations can be extended with content signatures, which can be introduced without having to replace existing identifier such as DOIs and ARKs. That is, signatures can be seen as complementary identifiers to help keep specific versions of cited data findable and identifiable as they evolve and change locations. For example, if a DOI identifies an evolving dataset, rather than a fixed version – i.e., content drift is expected – the DOI can safely be cited for the sake of attribution, metadata linking, and citation statistics (e.g., by Crossref (https://www.crossref.org) and DataCite (https://datacite.org)), while the content signature helps the reader find the exact content that was cited, possibly with assistance from metadata linked to the DOI. Additionally, a citation that includes both the DOI (for example) and content signature of a dataset creates a fixed mapping between the two identifiers. Then, unintentional content drift by the DOI can be detected and reported, and an alternative location may potentially be discovered by consulting public content signature registries.

Extending customary citation practices is not without challenges in conformability and interoperability with existing publisher workflows. Some publishers may not presently accept the inclusion of a content signature, perhaps alongside a DOI or other persistent identifier, whether due to incompatibilities with existing citation guidelines or document processing software. A discussion of how to address potential issues depends on the publisher and is beyond the scope of this paper.

### Cryptographic hashes are widely adopted

Our examples rely on the use of cryptographic hashing functions to form unique content signatures. As mentioned before, algorithms such as SHA-256 are not new, and have been widely used to secure digital communication channels (e.g., HTTPS/TLS), verify remote software updates^7^ (e.g., operating system upgrades), and in disaster recovery systems (e.g., automated backup systems^40^). More recently, cryptocurrencies such as Bitcoin and Ethereum make use of content hashes to record financial transactions in a decentralized network of independent servers (also known as miners). In addition, software to compute SHA-256 hashes is readily available from many trusted open-source projects and supported by most computer architectures (e.g., the sha25sum command in POSIX, the hashlib library in Python, the MessageDigest class in Java). Commands for calculating SHA-256 hashes in popular programming environments are listed in Table 1.

**Table 1:** Commands for calculating a SHA-256 hash in Bash, R, and Python shells.

Environment	Command
Bash*	$ sha256sum /some/path/bee-frontal.jpg
PowerShell	> Get-FileHash /some/path/bee-frontal.jpg
R	> install.packages(‘contentid’) > contentid::content_id(“/some/path/bee-frontal.jpg”)
Python	>>> import hashlib >>> content = open(‘/some/path/bee-frontal.jpg’, ‘rb’).read() >>> hashlib.sha256(content).hexdigest()

*The sha256sum command in the Bash example is part of GNU Coreutils.

### Readability of content signatures

Although our examples use SHA-256 content-hash URIs as content signatures, the content-hash URI specification (and others, such as named identifiers^24^) allows the use of other hash algorithms which may result in different hash lengths. Hash length is not a problem for machine readability and programmatic operations but can make it inconvenient for humans to read and write references. However, it is important to note that the strength of statistical uniqueness guarantees largely depends on hash length. We expect that data publishers and consumers will work together to find the most suitable compromise between uniqueness guarantees and readability. For instance, to shorten the 64 characters (that is, symbols such as letters and digits) needed to capture a SHA-256 hash in hexadecimal form, one can omit the second half of the hash at the cost of collision resistance (i.e., how difficult it is to find two pieces of content that result in the same hash^41^), reducing the number of possible hashes from the astronomical 2^256^ (about 10^77^, which is close an estimated number of atoms in the universe^42^) to a smaller but still galactic number, 2^128^.

64 characters: hash://sha256/29d30b566f924355a383b13cd48c3aa239d42cba0a55f4ccfc2930289b88b43c

32 characters: hash://sha256/29d30b566f924355a383b13cd48c3aa2

Even reducing the hash to 16 characters, the number of possible hashes is still 2^64^ (that is, 1.8 × 10^19^), more than the estimated number of cells in a human body^43^.

16 characters: hash://sha256/29d30b566f924355

Alternatively, more compact text encodings such as Base64^29^ can be used, but doing so can allow possibly confusing character combinations like the upper-case letter o, O, and zero, 0. Base64 identifiers may also include the symbols +, /, and =, which are commonly reserved as delimiters in URIs^28^ and therefore sometimes need to be percent-encoded (that is, encoding illegal characters as codes starting with %; e.g., URL encoding represents + as \%2B) when embedded within URIs such as URLs. This means that an identifier can have several possible Base64 representations depending on the need to use percent-encoding. Thus, Base64 introduces risks of misinterpretation by both human and machine users.

Long, unpredictable hashes may be especially susceptible to user transcription errors. If two people attempt to communicate a sentence verbally, or if one person attempts to manually write or type the sequence of characters that make up the words, spaces, and punctuation in a sentence, an observer can often detect errors in the received or transcribed text based on their understanding of word spellings and grammatical structures. However, cryptographic hashes typically have no predictable patterns by design. A user may check that a hash contains the expected number of characters and may be able to recognize suspicious sequences (01234, aaaaa, etc.) or illegal characters (standard hexadecimal representations may only include digits and the letters A through F), but it may be difficult to recognize whether a b was mistaken for a d, for example.

To detect transcription errors, a common and effective strategy is to use an error detection code, such as a checksum, hash, or parity bits^44^. Transcription errors can then be automatically detected by software by, for example, attempting to reproduce the error detection code. Some systems embed error detection codes within identifiers. For example, the International Bank Account Number (IBAN) system as defined by the International Organization for Standardization (ISO) includes two check digits in each bank account number which are determined – and can be verified – using the MOD97 algorithm. Error detection codes are also embedded in some content-based identifiers. The Named Identifiers for Humans (nih) specification^24^ allows the optional inclusion of a check digit alongside a hash, separated by a semicolon, where the check digit is calculated and verifiable by inputting the hash into Luhn’s mod 16 algorithm^24^. Although the URI specification^28^ does not support the embedding of error detection codes, the content-hash URIs we use can still be verified by recomputing the hash on the content it identifies, assuming the content is accessible. In the absence of the identified content, transcription can be made verifiable by providing check digits alongside content-hash URIs.

In this article, we primarily present content signatures as hexadecimal-encoded content-hash URIs in part due to our opinion that it is more easily understood than other available formats – they are proper URIs; they explicitly identify an algorithm as the identifier’s authority (e.g., sha256 is the URI authority component), rather than an organization, location, or nothing at all; they use only one character as a separator; and the namespace hash is descriptive, not an acronym – but also because it is the format is used in the real-world data featured in the examples in our Methods section. Among other alternatives mentioned in the Related Work section, it is worth noting that the named identifier (ni) scheme^24^ is currently under review by the Internet Engineering Task Force (IETF). However, syntactic differences in content signatures do not necessarily prevent interoperability across technical infrastructures. The uniquely identifying component of a content signature is the hash, whose bits remain the same regardless of how they are encoded as text. Thus, content signatures can be freely translated between different identifier schemes that support the same identifying semantics. For example, a hex-encoded content-hash URI can be translated as a Base64-encoded named identifier (ni) by decoding the hexadecimal representation of the hash, computing its Base64 representation, and decorating it with the expected syntax and semantics for named identifiers. The following two content signatures are semantically interchangeable:


hash://sha256/29d30b566f924355a383b13cd48c3aa239d42cba0a55f4ccfc2930289b88b43cni:///sha256;29d30b566f924355a383b13cd48c3aa239d42cba0a55f4ccfc2930289b88b43c


We also expect the development of text-processing tools that will be capable of translating between arbitrarily long content signatures and human-readable references in agreed-upon formats. Such tools may rely on mappings that could be part of the content that includes the references, or within metadata associated with the content. For example, a citation that includes both a DOI and a content signature explicitly links the two identifiers. By including these mappings within the content, human-readable references can be used for concise in-text citations while associated content-based identifiers enable verification and, if content-based resolution services are available, an alternative means of resolution to the cited data. In our supplementary document, we describe how existing software can be modified to use URLs as aliases for content signatures in order to create fixed associations between URLs and digital content.

### Content signatures enable independent content registries and repositories

In current content publication schemes, centralized entities take on the responsibility of maintaining the association between any identifier (e.g., a DOI) and the content it identifies (e.g., a publication). In the case of DOIs, a sophisticated software infrastructure is continuously maintained by a collection of dedicated institutions to make sure that the redirection of DOIs to their registered publishers works^45^. However, the registered publisher is solely responsible for maintaining the content at the location that a DOI resolves to. Although legal obligations concerning the management of resolved content may differ across Registration Authorities, in general nothing prevents a publisher from altering the located content, or in some cases even removing it entirely (e.g., DataCite’s online documentation states that DOIs should resolve to tombstones when content is lost or removed).

By using content-based identifiers such as content signatures, independent agents can use URLs or DOIs as temporary content aliases for more permanent content identifiers generated using a deterministic mathematical process. This process of feeding digital content into a cryptographic hashing function and recording its resulting hash can be performed at any location, by anyone, at any time, as long as the digital content and an implementation of the hashing function are at hand.

Because the content signature for any given digital file can always be regenerated, independent content lookup and retrieval services can be created to be interoperable without the need for a central registration infrastructure like the Handle System (for DOIs) or the Domain Name System (for URLs). These independent content services can further specialize without losing their independence. For instance, content signature registries (e.g., https://example-registry.org) can record associations of web locations (e.g., https://example-repository.org/photo.jpg) with references (e.g., hash://sha256/abc123…) to the content they provided at a specific point in time. These registries only have to store lightweight records (e.g., a timestamp, a URL, and a content signature) and leave the responsibility of storing the content to other specialized services: content repositories. These repositories only need to do one thing: provide the content associated with content signatures. With these independent content services, special knowledge networks can be engineered to register (or index) and/or store specific content. In other words, these knowledge networks can be designed to be decentralized. As an example of such a network, a Flickr-like service may offer a general image storage service, whereas a digital bee image library may keep only a registry of bee image signatures and the locations of their identified digital images across academic and commercial content repositories. Although such networks do not require centralized technical infrastructure, centralized organizational entities may assume responsibility in their respective communities for promoting interoperability standards, as well as social policies for protecting the network from data loss. At the same time, independent parties with interest in a particular knowledge network may also take measures to preserve information in the network. For example, national archives can be imagined that monitor valuable digital collections to ensure that a sufficient number of copies of each image remain available to minimize the risk of permanent data loss. In summary, content signatures allow for independent curation, storage, and discovery of digital collections of any kind of digital content.

In a content signature-based ecosystem, data providers (e.g., journals, individual researchers, autonomous measurement devices) store their data with one or more content repositories while advertising their content by informing one or more (independent) content registries of their storage locations. Archival services can partner with institutions to keep copies of relevant content (e.g., bee images) and register locations of the copies with independent content registries. Meanwhile, specialized entities can use registered content to provide specialized search services (demonstrated in our supplementary document). That said, digital (academic) libraries may choose to initially host their own services (e.g., content registries, repositories, and search engines) as well as collaborate with peers to maintain redundant or complementary infrastructures aimed at specific regional, national, or global use cases^37,46^. As content signature-based ecosystems grow and community members increasingly depend on particular services, community standards will be needed to ensure that key services remain available, protect communities from data loss, and encourage consistent content identification practices.

Some academic data publication infrastructures (e.g., Zenodo, DataONE, Software Heritage) already allow some content to be located using an associated hash. For instance, users can search for data publications on Zenodo that contain files matching an MD5 hash (although this functionality is not officially supported). Similarly, DataONE and the Software Heritage archive allow users to query their data registries for content using SHA-256 hashes. Software Heritage even forms content signatures using their own URI-conforming identifier scheme, Software Heritage Persistent ID^25^ (SWH-ID), for which there are several online resolvers (e.g., https://archive.softwareheritage.org). However, to our knowledge, these infrastructures do not verifiably and recursively link content directly to each other by their content signatures, nor do they provide discovery services for these types of links.

To facilitate interoperability between services that present their content signatures using different identifier schemes, a service that receives content signatures as input might choose to translate them to their preferred representation for their internal processes. However, hashes produced by different hashing algorithms are not interchangeable. For example, an MD5 content-hash used by Zenodo cannot be translated into a SHA-1 SWH-ID used by the Software Heritage archive.

### Data management plans and keeping track of project data outputs

Funders of academic research increasingly support open science principles to democratize access to research outcomes. To help facilitate this, data management plans are required for some grant proposals. These plans capture how a researcher plans to keep and publish their data. To help implement these plans, the digital content produced by a project can be tracked over time and packaged into citable, publishable units (as shown in example 3 and in our supplementary document) using tools like Preston. Not only can this help researchers and funders more easily share structured progress reports, it also exercises their ability to create versioned data publications linked over time. Finally, having a location-agnostic method to independently track progress of research data opens up the possibility to be flexible on where to (temporarily) store and relocate copies of valuable scholarly assets over time.

### Similar, but not the same: dealing with similar copies

**Fig. 3:**
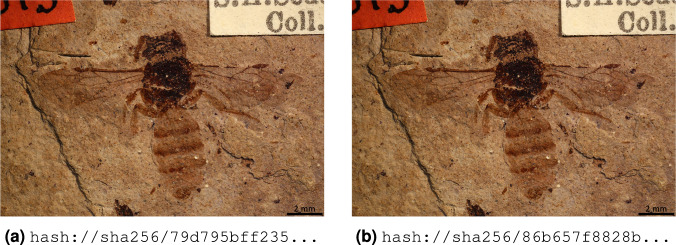
Renderings of two digital images of *Andrena sepulta* (Cockerell, 1906) extracted from A Biodiversity Dataset Graph: https://jhpoelen.nl/bees. 2020. hash://sha256/85138e506a29fb73099fb050372d8a379794ab57fe4bfdf141743db0de2b985c. The depicted image of a wing is used with permission from the Museum of Comparative Zoology, Harvard University, ©President and Fellows of Harvard CC-BY-NC-SA 4.0. The image was not modified from its original form.

Digital data are often transformed or altered. The resulting changes can be intentional or accidental side effects of manual or automated data processes, and can sometimes be hard for humans to detect. For instance, the two bee images in Fig. 3 are visually indistinguishable even though their binary contents are very different, such that a piece of software may succeed in processing one while failing with the other. The differences may originate from a change in resolution, image format conversion, or a change of a single bit in the original digital file due to a copy error. Even though a human might not be able to notice any difference when examining renderings of the images, the difference in their content signatures is easy to catch.

The images in Fig. 3 capture digital photos of a specimen referenced by the URL http://mczbase.mcz.harvard.edu/guid/MCZ:Ent:PALE-2013 and described in Cockerell, 1906^47^. Image A was retrieved from https://api.idigbio.org/v2/media/496d3050-b7cc-4ff0-a45c-bc524e0b7f9d?size = fullsize with content signature hash://sha256/79d795bff23567be10d36934facc8befca300e426d0abbd7c273269fe8ec427b. Image B was retrieved from http://mczbase.mcz.harvard.edu/specimen_images/entomology/paleo/large/PALE-2013_Andrena_sepulta_holotype.jpg with content signature hash://sha256/86b657f8828b235371322894c65ec62909f62f874c1b1528e49edddb5fc2271a.

Even if a human is able to notice the difference, we still need a reliable method to establish that the files are different. After determining that files are different, human observers may have an opinion on the relevance of the difference: for some observers, the two images may carry the same meaning, whereas others might want to treat the images as distinct pieces of photographic evidence. With content signatures, we have a robust way to express statements like Image A and image B are the same or Image A and image B are different because we can unambiguously reference image A and image B and remove any kind of confusion about some equivalence claim of digital content, regardless of their type (e.g., text, image, sound) or format (e.g., ASCII, JPEG, WAV). A structured machine-readable format (e.g., RDF) may be used to express these opinions about the images, but natural languages (e.g., English, Chinese) can also be used to make reasonably precise, accurate, and verifiable statements about these images.

A similar argument can be made for digital texts and structured-data documents (e.g., XML, JSON, HTML files): digital texts may look identical to humans even if their digital contents are different. These changes may originate from cosmetic differences (e.g., “hello[space]world” vs. “hello[space][space]world”, “<b>hello world</b>” vs. “hello world”), but can also include more complex differences (e.g., “I ate spinach with a fork” vs. “I used a fork to eat spinach”). Our method aims to preserve the reference to the original digital assets without imposing any opinions or restrictions beyond the choice of cryptographic hashing function. Still, statements can be published to record opinions about relations between distinct digital assets. Then, these recorded opinions can be carefully reviewed or otherwise taken into account. In other words, by using objective methods to reference digital data and related statements, claims and their provenance (or origination) can be more precisely recorded.

Similar considerations can be made in relation to implicit orderings of content within a collection. Such an ordering is implied by the computation of the hash embedded in a content signature. For example, the hash of {a,b} is different from the hash of {b,a}. However, one cannot state in general that the two sets should or should not be uniquely identified as being either the same or different. In practical situations, for domains where sets are identical regardless of ordering, it may be possible to compute hashes using canonical set representations where, for example, the set members are lexicographically ordered. In such a case, the computation of signatures would require a preprocessing step to generate the canonical representation (e.g., lexicographically sorting the set members), followed by the computation of the hash.

Perhaps needless to say, relations between distinct digital objects can be recorded individually by humans following visual inspection, but automated processes can also be used. We imagine that computer algorithms (e.g., text normalization, deduplication, image classification) can be used to quickly and automatically generate claims about how specific data relate to each other. More generally, locality-sensitive hashing techniques^48,49^ have been developed to generate semantic hashes or fuzzy hashes which can be compared to detect similarities (as defined by the algorithm) between their associated digital contents. A familiar example is YouTube’s Content ID, which uses semantic hashes (which they call digital fingerprints) to detect copyrighted video and audio segments – including derivative content, such as a fan cover of a popular song – in videos submitted to YouTube. Clearly, however, YouTube’s goal is to distinguish original content from the unoriginal, and therefore other identifiers are used alongside semantic hashes to uniquely identify content. While semantic hashes can be used to make similarity claims about digital contents, they are ill-suited as unique identifiers due to their subjectivity (different algorithms will generate different similarity claims), sensitivity (an algorithm can only meaningfully capture similarities based on specific data patterns for which it was designed), and, for many algorithms, poor collision resistance (non-identical contents may be assigned the same hash).

### Signatures enhance citation persistence

A citation is persistent if it is resolvable to a location of the cited object, mechanisms (social or technical) exist to ensure future (though not necessarily indefinite) resolvability, and it never resolves to a location containing anything that could be misidentified as the cited object. In a parallel publication, we show that identifiers that are unique, verifiable, and location-agnostic require less investment to ensure the continued and consistent resolvability required for citation persistence. We have discussed identifier verifiability at length in previous sections, so in this discussion we focus on uniqueness and location-agnosticism.

An identifier is unique if it is assigned to only one object and is never reused^27^. Cryptographic content hashes of sufficient length can algorithmically guarantee uniqueness without any required coordination effort^7^. In contrast, non-content-based identifiers such as DOIs tend to rely on public infrastructures and coordinated community efforts to orchestrate the unique minting of new identifiers and to maintain up-to-date registries of the locations of identified objects^21^. Although non-content-based identifier specifications such as Universally Unique Identifier (UUID) version 4 (assuming a truly random number generator is used^50^) may also statistically guarantee that an identifier is never generated twice, they are unverifiable, and therefore require an intermediary (i.e., infrastructure) to maintain unique object identifier assignments.

An identifier that is location-agnostic allows the identified object to be moved without losing persistence. Specifically, this means that resolution of the identifier to one or more known content locations can be fully reconfigurable. Different identifier schemes impose different constraints on resolution reconfigurability, often due to embedding location or ownership information within the identifier. Location-based identifiers such as URLs may allow limited reconfigurability (provided the owner of the URL’s domain name is willing and the URL has not been repurposed) by implementing redirects to alternative locations or changing mappings between location names and physical locations, e.g., domain names and Internet Protocol addresses in the Domain Name System. DOI reconfiguration similarly relies on redirects, though authority to implement them rests with designated rights holders, who are expected to maintain identifier persistence^45^, rather than with Internet domain name owners, who are not. Purely content-based hashes always allow resolution reconfiguration; because hashes are not bound to any one infrastructure or organization, anyone is free to set up registries and resolution services.

Accepting that content signatures are unique, verifiable, and location-agnostic, the persistence that they enable for signed citations may only be expected with sufficient community adoption; registries must be publicly accessible and up to date with the locations of content stored across independent content repositories, and readers must be aware of how to recognize signed citations and resolve their content signatures. In lieu of widespread adoption, content signatures can be included alongside other identifiers to provide verifiability for citations that rely on well-established yet ordinarily non-verifiable identifier schemes and resolution services. Content signatures may even be embedded within other identifiers, such as DOIs, to impart verifiability and guarantee uniqueness while retaining restrictions on resolution reconfigurability.

### Challenges for community adoption

We acknowledge that community adoption of content signatures poses sociotechnical challenges. Several such challenges are discussed at greater length in earlier discussion sections. In the “Extending customary citation practices” discussion, we consider content signatures to be complementary to existing persistent identifiers, such as DOIs, although publishers may have citation guidelines that do not permit multiple identifiers or unrecognized identifier schemes. In “Readability of content signatures”, we discuss the need for community standards regarding content signature readability, interpretability, and interoperability. “Content signatures enable independent content registries and repositories” imagines the incremental development of independent signature-based services that work with existing infrastructures rather than replacing them. The need for community coordination to ensure data persistence is highlighted in “Content signatures enable independent content registries and repositories” and “Signatures enhance citation persistence”.

Because the computation and resolution of content signatures do not rely on centralized administration, communities are free to independently advance their own standards and services. Thus, we encourage communities that may benefit from signed citations to consider standards that meet their specific needs, rather than attempting to find solutions that work for everyone. Communities need only interact with their preferred, domain-specific data publishers to reach consensus on the use of content signatures in citations. To some extent, this is already happening. As proposed in^22^, the Dataverse citation guidelines (available at https://guides.dataverse.org) suggest including universal numerical fingerprints in citations alongside persistent identifiers. Meanwhile, Software Heritage advocates for the inclusion of SWH-IDs in software citations, as demonstrated in^25^. When the separate inclusion of content signatures in citations does not comply with publisher citation guidelines, they may still be embedded in other identifiers, such as URLs, as^23^ suggests for Trusty URIs. Note that the adoption of community-centric citation standards does not bar others from referencing community data using other methods. More general services such as content signature registries are always free to externally catalog content across communities without requiring special accommodations.

## Conclusions

Signed citations offer a verifiable method of citing digital content in a way that robustly links data to form complex, verifiable, and citable citation graphs. The use of verifiable citations and citation graphs can contribute to the reproducibility of data-backed research by facilitating continued access to the data as originally cited, and preventing the accidental use of the wrong data. In our experience with applying signed citations to real world data-oriented problems, we have found that content signatures scale well, allow for building independent and decentralized content services, and can be introduced without disrupting existing publication systems and identifier infrastructures. Furthermore, we have described how – compared to unverifiable identification methods – signed citations can require less investment to maintain their persistence. Although some existing online platforms such as Zenodo and the Software Heritage archive can be leveraged for proof-of-concept content signature-based registry, discovery, and retrieval services, there is ample opportunity for the development of new signature-based services (e.g., registries, specialized search indexes, automated signature verification monitors), further integration of content signatures into existing online platforms, and the development of community standards (e.g., preferred hash algorithms and lengths, hash encoding methods, embedding hashes within DOIs, data provenance requirements, and best practices and coordinated efforts for preventing data loss) needed to encourage interoperability across independent services. Even without a developed ecosystem of standards and services, content signatures can still be included in citations of digital content – as publishers permit – to reinforce them with verifiability and a robust option for future resolvability, for both packaged content and distributed content networks. Our work suggests that signing citations helps make our digital academic knowledge more resilient against World Wide Web phenomena like link rot and content drift in an ever-changing technology landscape. Therefore, we ask you to consider signing your digital data citations and help preserve our digital heritage.

## Supplementary information

Supplementary Information

## Data Availability

The Bees biodiversity dataset graph^35^ analyzed in the examples is available in Zenodo at 10.5281/zenodo.7036080. The Zenodo publication contains the graph cited in the examples with content signature hash://sha256/85138e506a29fb73099fb050372d8a379794ab57fe4bfdf141743db0de2b985c. The publication can also be found by entering the content signature’s hash into the search bar at https://zenodo.org.

## Code availability

The source code for the Preston software^32^ is available in GitHub at https://github.com/bio-guoda/preston and in Zenodo at 10.5281/zenodo.1410543. Preston version 4.4 has been assigned the 10.5281/zenodo.7005141. The MD5 content signature for the zip archive of the source code is hash://md5/5339621a0b95bfbbf450063d76057d2f.

## References

[1] Comer, D. E. *Internetworking with TCP/IP, Volume 1: Principles, Protocols, and Architectures*, 4th edn (Prentice Hall PTR, USA, 2000).

[2] Klein M (2014). Scholarly Context Not Found: One in Five Articles Suffers from Reference Rot. PLoS ONE.

[3] Kunze, J. & Rodgers, R. The ARK Identifier Scheme. Tech. Rep., UC Office of the President: California Digital Library. https://escholarship.org/uc/item/9p9863nc (2008).

[4] Paskin N (2010). Digital Object Identifier (DOI) System. Encyclopedia of Library and Information Sciences.

[5] Elliott MJ, Poelen JH, Fortes JAB (2020). Toward reliable biodiversity dataset references. Ecological Informatics.

[6] Dang QH (2015). Secure hash standard. Tech. Rep., National Institute of Standards and Technology..

[7] Sobti R, Geetha G (2012). Cryptographic Hash Functions: A Review. International Journal of Computer Science Issues (IJCSI).

[8] Primmer, R. & D’Halluin, C. Collision and Preimage Resistance of the Centera Content Address. *CoRR***abs/1306.6020**, 10.48550/arXiv.1306.6020 (2013).

[9] Dilley J (2002). Globally distributed content delivery. IEEE Internet Computing.

[10] Koponen T (2007). A Data-Oriented (and beyond) Network Architecture. ACM SIGCOMM Computer Communication Review.

[11] Dannewitz C (2013). Network of Information (NetInf) – An information-centric networking architecture. Computer Communications.

[12] Tarr, D., Lavoie, E., Meyer, A. & Tschudin, C. Secure Scuttlebutt: An Identity-Centric Protocol for Subjective and Decentralized Applications. In *Proceedings of the 6th ACM Conference on Information-Centric Networking*, **ICN ‘19**, 1–11, 10.1145/3357150.3357396 (Association for Computing Machinery, New York, NY, USA, 2019).

[13] Nakamoto, S. Bitcoin: A peer-to-peer electronic cash system. Accessed at https://bitcoin.org/bitcoin.pdf (2008).

[14] Nour, B., Khelifi, H., Hussain, R., Mastorakis, S. & Moungla, H. Access Control Mechanisms in Named Data Networks: A Comprehensive Survey. *ACM Computing Surveys* **54**, 10.1145/3442150 (2021).

[15] Rathod, U., Sonkar, M. & Chandavarkar, B. R. An Experimental Evaluation on the Dependency between One-Way Hash Functions and Salt. In 2020 *11th International Conference on Computing, Communication and Networking Technologies (ICCCNT)*, 1–7, 10.1109/ICCCNT49239.2020.9225503 (2020).

[16] Poelen JH, Simons JD, Mungall CJ (2014). Global biotic interactions: An open infrastructure to share and analyze species-interaction datasets. Ecological Informatics.

[17] Stanisic, L. A Reproducible Research Methodology for Designing and Conducting Faithful Simulations of Dynamic HPC Applications. *Theses, Université Grenoble Alpes*. Accessed at https://theses.hal.science/tel-01248109 (2015).

[18] Dillen, M., Groom, Q., Agosti, D. & Nielsen, L. H. Zenodo, an Archive and Publishing Repository: A tale of two herbarium specimen pilot projects. *Biodiversity Information Science and Standards*10.3897/biss.3.37080. Article (2019).

[19] Di Cosmo, R. & Zacchiroli, S. Software Heritage: Why and How to Preserve Software Source Code. In *iPRES 2017 - 14th International Conference on Digital Preservation*, 1–10. Accessed at https://hal.science/hal-01590958 (Kyoto, Japan, 2017).

[20] Zheng Z, Xie S, Dai H-N, Chen X, Wang H (2018). Blockchain challenges and opportunities: a survey. International Journal of Web and Grid Services.

[21] Hakala, J. Persistent identifiers - an overview. *KIM Technology Watch Report* (2010). Accessed at https://www.semanticscholar.org/paper/Persistent-identifiers-an-overview-Hakala/2c679447c394b59e095b3ef184f6e1c0f1be97fc.

[22] Altman, M. & King, G. A proposed standard for the scholarly citation of quantitative data. *D-lib Magazine* **13**. Accessed at https://ssrn.com/abstract=1081955 (2007).

[23] Kuhn, T. & Dumontier, M. Trusty uris: Verifiable, immutable, and permanent digital artifacts for linked data. In Presutti, V. et al. (eds.) *The Semantic Web: Trends and Challenges*, 395–410, 10.1007/978-3-319-07443-6_27 (Springer International Publishing, Cham, 2014).

[24] Farrell S (2013). Naming Things with Hashes. Tech. Rep. 6920, RFC Editor..

[25] Di Cosmo, R. Archiving and referencing source code with software heritage. In Bigatti, A. M., *et al* (eds.) *Mathematical Software–ICMS 2020*, 362–373, 10.1007/978-3-030-52200-1_36 (Springer International Publishing, Cham, 2020).

[26] Golodoniuc, P., Car, N. N. J. & Klump, J. Distributed Persistent Identifiers System Design. Data Science Journal **16**, 10.5334/dsj-2017-034 (2017).

[27] Paskin, N. Toward unique identifiers. *Proceedings of the IEEE* **87**, 1208–1227, 10.1109/5.771073 (1999).

[28] Berners-Lee T, Fielding RT, Masinter LM (2005). Uniform Resource Identifier (URI): Generic Syntax. Tech. Rep. 3986, RFC Editor..

[29] Josefsson S (2006). The Base16, Base32, and Base64 Data Encodings. Tech. Rep. 4648, RFC Editor..

[30] Gorn S, Bemer RW, Green J (1963). American Standard Code for Information Interchange. Communications of the ACM.

[31] Lebo, T., Sahoo, S. & McGuinness, D. PROV-O: The PROV Ontology. W3C Recommendation, W3C. Accessed at https://www.w3.org/TR/2013/REC-prov-o-20130430/ (2013).

[32] Poelen JH, Elliott MJ, Alzuru I (2023). Zenodo.

[33] Carothers, G. *RDF 1.1 N-Quads. W3C Recommendation, W3C.* https://www.w3.org/TR/2014/REC-n-quads-20140225/ (2014). Accessed at.

[34] Harris, S. & Seaborne, A. SPARQL 1.1 Query Language. W3C Recommendation, W3C. Accessed at https://www.w3.org/TR/2013/REC-sparql11-query-20130321/ (2013).

[35] Poelen J (2022). Zenodo.

[36] Poelen JH, Elliott MJ (2021). Zenodo.

[37] C. Seltmann K (2021). Announcing Big-Bee: An initiative to promote understanding of bees through image and trait digitization. Biodiversity Information Science and Standards.

[38] Poelen J, Salim J (2022). Zenodo.

[39] Poelen JH (2022). Zenodo.

[40] Liu, J., Tan, Y., Li, Y., Zhang, X. & Zhou, Z. A Method of Deduplication for Data Remote Backup. In Li, D., Liu, Y. & Chen, Y. (eds.) *Computer and Computing Technologies in Agriculture IV*, 68–75, 10.1007/978-3-642-18333-1_10 (Springer Berlin Heidelberg, Berlin, Heidelberg, 2011).

[41] Girault, M. & Stern, J. On the length of cryptographic hash-values used in identification schemes. In Desmedt, Y. G. (ed.) *Advances in Cryptology–CRYPTO ‘94*, 202–215, 10.1007/3-540-48658-5_21 (Springer Berlin Heidelberg, Berlin, Heidelberg, 1994).

[42] Broad CD (1940). Sir Arthur Eddington’s The Philosophy of Physical Science. Philosophy.

[43] Bianconi, E. et al. An estimation of the number of cells in the human body. *Annals of Human Biology* **40**, 463–471, 10.3109/03014460.2013.807878. PMID: 23829164 (2013). 10.3109/03014460.2013.807878 23829164

[44] Moreira, J. C. & Farrell, P. G. *Essentials of Error-Control Coding* (John Wiley & Sons, Chichester, England, 2006).

[45] The International DOI Foundation. DOI Handbook, 10.1000/182 (2022).

[46] (2021). Zenodo.

[47] Cockerell, T. D. A. Fossil Hymenoptera From Florissant, Colorado. *Bulletin of the Museum of Comparative Zoology at Harvard College* **50**, 3–58. Accessed at https://digitalcommons.usu.edu/bee_lab_ca/356 (1906).

[48] Gionis, A., Indyk, P. & Motwani, R. Similarity Search in High Dimensions via Hashing. In Proceedings of the 25th International Conference on Very Large Data Bases, VLDB ‘99, 518–529. Accessed at https://dl.acm.org/doi/10.5555/645925.671516 (Morgan Kaufmann Publishers Inc., San Francisco, CA, USA, 1999).

[49] Salakhutdinov, R. & Hinton, G. Semantic hashing. *International Journal of Approximate Reasoning* **50**, 969–978, 10.1016/j.ijar.2008.11.006. Special Section on Graphical Models and Information Retrieval (2009).

[50] Leach PJ, Salz R, Mealling MH (2005). A Universally Unique IDentifier (UUID) URN Namespace. Tech. Rep. 4122, RFC Editor..

