# Supplementary material for: Signing data citations enables data verification and citation persistence
Source: Sci Data. 2023 Jun 27;10:419. doi: 10.1038/s41597-023-02230-y (PMC10300068; doi:10.1038/s41597-023-02230-y)
Supplement: Supplementary file 1 — Supplementary Information [file 41597_2023_2230_MOESM1_ESM.pdf]

Signing data citations enables data verification and citation persistence - Supplementary Material

Michael J. Elliott[1,†], Jorrit H. Poelen[2,3,†], José A.B. Fortes[1]

[1] University of Florida, Gainesville, United States of America

[2] Ronin Institute, Montclair, NJ, United States of America

[3] UC Santa Barbara Cheadle Center for Biodiversity and Ecological Restoration, Santa Barbara, CA, United States of America

[†] These authors contributed equally to this work

Contents

1. Compiling the distributed UCSB CCBER image corpus 1

2. Versioning, citing, and retrieving biodiversity datasets registered With GBIF, iDigBio, and BioCASe 2

3. Stabilizing URL references using Nomer's Corpus of Taxonomic Resources 3

4. Our use of Zenodo as a proof-of-concept content signature resolver 4

5. Enhancing existing content discovery and retrieval methods with signed citations 5

References 7

1. Compiling the distributed UCSB CCBER image corpus

The National Science Foundation has recently funded an ambitious project named "Extending Anthophila research through image and trait digitization (Big-Bee)" (Seltmann et al., 2021). The project aims to "create over one million high-resolution 2D and 3D images of bee specimens." A challenge for the project is to keep track of, and reference, a growing corpus of images in a distributed network of participating institutions and natural history collections. Over the course of the project, the image corpus is expected to grow vertically by adding more images to the Zoology Collection at UCSB, but also horizontally by including additional participating institutions. As the Big-Bee corpus grows, we plan to separately track the individual collections and keep an aggregated index to robustly link the image corpora. This way, individual institutions have the flexibility to keep copies of their image in preferred locations, while the overarching project can reliably reference and access the results of their combined efforts.

Conventional citation methods may, for example, require packaging the entire corpus as a single archive with an assigned DOI. This is undesirable for the Big-Bee corpus (which is expected to grow to one million images), as the size of the archive may be prohibitively large for some users, and

expensive to preserve older versions as new versions are published. Alternatively, each image may be assigned its own DOI, requiring users to cite images individually or to cite a collection description that in turn cites each image by its DOI. This case demands a considerable amount of effort and commitment beyond the duration of the project to maintain active links (e.g., URLs) from each DOI to its associated image.

Instead, we make the Big-Bee corpus citable by publishing a registry of content signatures for images and associated metadata (including the locations of the images), such that a signed citation of the registry (10s of megabytes) recursively and robustly cites the entire Big-Bee image collection (potentially 100s of gigabytes). After accessing the registry, users can choose to locate and retrieve the subset of images they are interested in. When new images become available or change locations, the registry can be updated by publishing a new version that cites the previous version and appends the new information. Thus, snapshots of the Big-Bee collection may be cited and subsequently retrieved at any time without requiring any further commitment by the Big-Bee participants to maintain earlier versions of the collection (note that successive versions only add new images, and do not remove existing images). Additionally, when attempting to retrieve cited images, future versions of the registry (or even other independent registries) may be consulted to locate images that have changed locations but retained their unique content signatures.

Below, two versions of the Big Bee image corpus are cited using a single signature. Because the second version cites, contains, and extends the first version, only the content signature of the second version needs to be included in the citation. At least three copies of the corpus are kept at the Internet Archive, Zenodo, and a collection management system at the University of California at Santa Barbara's Cheadle Center for Biodiversity and Ecological Restoration (UCSB CCBER).

```
Cheadle Center for Biodiversity and Ecological Restoration, University of
California Santa Barbara. (2021). UC Santa Barbara Invertebrate Zoology
Collection (UCSB-IZC) Data Archive and Biodiversity Dataset Graph (0.2) [Data
set]. Zenodo. https://doi.org/10.5281/zenodo.
hash://sha256/80c0f5fc598be1446d23c95141e87880c9e53773cb2e0b5b54cb57a8ea00b20c
```

2. Versioning, citing, and retrieving biodiversity datasets registered With GBIF, iDigBio, and BioCASe

Recursive signed citations were used to construct the Biodiversity Dataset Archive (Poelen & Elliott, 2021). The archive contains openly available biodiversity datasets from institutions around the world as listed by dataset registries hosted by GBIF (https://gbif.org), iDigBio (https://idigbio.org), and BioCASe (https://biocase.org). The archive also contains machine-readable provenance logs (generated using the Preston tool, much like example 3) that describe the discovery of registered biodiversity dataset URLs, attempts to retrieve datasets from those URLs, and the content signatures of all collected datasets and provenance logs. The information in the provenance logs forms a graph consisting of links between URLs, datasets, automated discovery and collection processes, and intermediate data collected by those processes (e.g., the dataset registries hosted by GBIF, iDigBio, and BioCASe), where all data are referenced using their content signatures. Consequently, all digital resources (datasets, provenance logs, and intermediate outputs of automated processes) in the graph

are directly or indirectly referenced by the latest provenance log, such that a citation (e.g., example 3) of the latest provenance log recursively cites the entire graph and everything referenced by it. The graph was analyzed in Elliott et al. 2020 to collect statistics on how often the availability and content of biodiversity dataset URLs change over time, as indicated by changes in the content signatures associated with each URL in response to monthly download requests.

The citation of the large and complex data graph containing billions of versioned biodiversity records published independently across hundreds of institutions is similar to citing a single image. Below is a signed citation of a graph of biodiversity datasets registered with GBIF, iDigBio, and BioCASe. This is the 50th version of an evolving graph; it cites, contains, and extends 49 earlier versions. Rather than computing a hash of the entire Z TB archive, we only need to compute the hash of the latest provenance log, which recursively and verifiably cites all other data in the graph by their SHA-256 content signatures.

```
Poelen, J.H., Elliott, M.J. (2020). Biodiversity Dataset Archive.
Hash://sha256/8aacce08462b87a345d271081783bdd999663ef90099212c8831db399fc0831b
Accessed at https://archive.org/details/biodiversity-dataset-archives
```

The exact version of the graph referenced in the citation can be located by entering the content signature into the search bar at zenodo.org (note that we had to indicate to Zenodo that this specific SHA-256 hash should be included in their search index). The graph (distributed across several provenance datasets) and the content it references can then be downloaded manually or using an automated tool such as Preston (Poelen et al., 2023). Once downloaded, the signatures of the data in the archive – as described in the provenance logs, starting with the one that was cited – may be reproduced using the SHA-256 algorithm to verify that the correct data has been retrieved.

3. Stabilizing URL references using Nomer’s Corpus of Taxonomic Resources

Existing compute infrastructures and data processing software often use URLs to reference digital data. For instance, Nomer (Poelen & Salim, 2022) is a tool to help interpret taxonomic names using existing taxonomic resources (e.g., NCBI Taxonomy (Federhen, 2012)). Nomer uses URLs to point to externally published taxonomic resources (e.g., https://ftp.ncbi.nlm.nih.gov/pub/taxonomy/taxdump.tar.gz). Because the content accessed via these URLs may change or become unavailable over time, the results produced by Nomer may change over time. To help make Nomer’s results reproducible over time, the Nomer Corpus of Taxonomic Resources was created (Poelen, 2022). By using principles of content addressing in the form of content signatures, this corpus associates locations of taxonomic resources with their resolved digital content at several different observation times. Nomer has been extended to look up previously resolved content associated with a location as defined in a specific version of the taxonomic resource corpus to ensure that Nomer’s results remain reproducible, even as the availability of taxonomic resources at their original Internet locations changes over time. Nomer's Corpus of Taxonomic Resources is cited below.

```
Poelen, J.H. (2021). Nomer Corpus of Taxonomic Resources.
hash://sha256/bb6dac6461b66212c5b1826447d7765529ff5cbadeac1915f7c3be9748eda991
(0.1) [Data set]. Accessed at https://doi.org/10.5281/zenodo.5639794
```

We expect that having a reliable method to interpret taxonomic names will help to better understand and quantify the impact of different taxonomic interpretations on research outcomes. For instance, by directing Nomer to use a mapping of URLs to content signatures at a specific point in time, we are able reproduce Nomer's recognition of previously unknown differences in bee names between the specified versions of ITIS (ITIS 2004) and the Discover Life bee species guide and world checklist (Ascher & Pickering 2020).

4. Our use of Zenodo as a proof-of-concept content signature resolver

Our use of Zenodo for resolving MD5 and SHA-256 hashes to content made use of two unsupported features, and should not be expected to work as expected without official support. For resolving MD5 hashes, it was demonstrated in an online forum on Zenodo's GitHub project page (https://github.com/zenodo/zenodo/issues/1985) that Zenodo's search API allows users to query for content that match a supplied MD5 hash. The API can be used in this way by forming a URL with the following pattern:

```
https://zenodo.org/search?q=_files.checksum:"[MD5 hash]"
```

For example,

```
https://zenodo.org/search?q=_files.checksum:"md5:898a9c02bedccaea5434ee4c6d64b
7a2"
```

Because the web API uses a predictable format, users and automated services can effectively (with the understanding that it is unsupported) use Zenodo as an MD5 resolver for any content that they serve.

We also used Zenodo as a resolver for SHA-256 hashes, but using a different approach. Because Zenodo indexes the contents of each publication's title and readme, any hashes (preferably with the algorithm specified, e.g., using "content signatures") included in those fields will be made findable by their search API. For example, the following query reliably returns a link to a dataset identified by the content signature hash://sha256/450deb8ed9092ac9b2f0f31d3dcf4e2b9be003c460df63dd6463d252bff37b55.

```
https://zenodo.org/search?q=450deb8ed9092ac9b2f0f31d3dcf4e2b9be003c460df63dd64
63d252bff37b55
```

Because the dataset's MD5 hash is also listed in the readme, the dataset can also be found by its MD5 hash using this method:

```
https://zenodo.org/search?q=898a9c02bedccaea5434ee4c6d64b7a2
```

In general, any online content repository with a web API that allows searching by hash, and that produces predictably formatted outputs, can also act as a content resolver, usually with the restriction that only hashes of content housed in the content repository will be resolvable. In light of this, to help users resolve content, the Preston software simply queries several different repositories rather than requiring the user to specify which resolver (i.e., content signature registry) to use.

5. Enhancing existing content discovery and retrieval methods with signed citations

Content discovery (searching for data) and content retrieval (getting the data) methods are needed to make use of available digital content. A typical content discovery and retrieval use case can be exemplified as follows:

1. Dr. Yasmine Abbas articulates her search request and sends it to José Martinez, a trusted library agent.
2. After receiving and processing Dr. Abbas' request, Martinez compiles a list of relevant scholarly publications.
3. Dr. Abbas retrieves the first listed digital resource from the institutional digital archive.
4. After using the digital resource in her work, Dr. Abbas includes a citation to the resource in her reference list.

This sequence of steps can be robustly executed, documented, linked to the resulting data using signed citations as follows. In step 1, Dr. Abbas creates a search request, and then submits it to a library service. This search criteria is a small publication by Dr. Abbas that documents her interest. Let's say her request is captured in the following text document:

```
Dr. Abbas requests references to digital images of the Bee genus Apis.
```

In step 2, Dr. Abbas receives the following result compiled by the library agent, Martinez,

```
In response to "Dr. Abbas requests references to digital images of the Bee genus
Apis", Jose Martinez located the following images after consulting "A
biodiversity dataset graph: https://jhpoelen.nl/bees. 2020.
hash://sha256/85138e506a29fb73099fb050372d8a379794ab57fe4bfdf141743db0de2b985c":

Museum of Comparative Zoology, Harvard University. 2021. Head Frontal View of
MCZ:ENT:17219 Nomadopsis puellae (Cockerell, 1933)
hash://sha256/edde5b2b45961e356f27b81a3aa51584de4761ad9fa678c4b9fa3230808ea356 .
Accessed at http://mczbase.mcz.harvard.edu/specimen_images/entomology/large/MCZ-
ENT00017219_Spinoliella_puellae_hef.jpg on 2021-12-07.

Museum of Comparative Zoology, Harvard University. 2021. Habitus Lateral View of
MCZ:ENT:17219 Nomadopsis puellae (Cockerell, 1933)
hash://sha256/8d49bd24f6ba300b4de44fd218b53294f4cc0106cd9631018ef819b38345c75d.
Accessed at http://mczbase.mcz.harvard.edu/specimen_images/entomology/large/MCZ-
ENT00017219_Spinoliella_puellae_hal.jpg on 2021-12-07.
```

along with a signed citation for the list,

```
List of references to digital images of the Bee genus Apis compiled by Jose
Martinez in response to Dr. Abbas’ request:
hash://sha256ca712a49029b4d956d1dbf4b32174ce2297d362118464b057d5d66afbc99767c
```

where the hash of Dr. Abbas’ request is included to verifiably link the Martinez’ response to Dr. Abbas’ request. Note that Martinez explicitly states which resource (a biodiversity dataset graph of bee-related records) was used to generate the list. If desired, Dr. Abbas can review and verify his results.

In step 3, Dr. Abbas sends another request to Martinez:

```
Please send me the *digital content* related to: Museum of Comparative Zoology,
Harvard University. 2021. Head Frontal View of MCZ:ENT:17219 Nomadopsis puellae
(Cockerell, 1933)
hash://sha256/edde5b2b45961e356f27b81a3aa51584de4761ad9fa678c4b9fa3230808ea356.
Accessed at http://mczbase.mcz.harvard.edu/specimen_images/entomology/large/MCZ-
ENT00017219_Spinoliella_puellae_hef.jpg on 2021-12-07.
```

After receiving the request, Martinez sends an exact digital copy of the requested content to Dr. Abbas. On receiving the digital content, Dr. Abbas successfully verifies the identity of the content by calculating its content hash and confirming that it matches the hash cited in her request. In step 4, Dr. Abbas uses the digital content in a publication, and includes a reference to the content in her list of references.

Now that we've described a content discovery and retrieval scenario, we can articulate the benefits of incorporating content signatures into discovery and retrieval workflows: signatures allow data and their provenance to be reliably described so that, if desired, Dr. Abbas can independently verify the claims made by Martinez. The use of content-based identifiers allows the discovery and retrieval method to be independent of communication protocol, location, and time:

1. Communication protocol independent – regardless of the communication method or storage medium, Dr. Abbas is able to verify the identity of the retrieved content by recalculating its signature. Also, Dr. Abbas is able to check whether Martinez correctly referenced her original request. In addition, Dr. Abbas can trace the provenance of the (possibly) subjective statements made by Martinez. In the absence of verifiable references such as content signatures, Dr. Abbas has to trust, but cannot verify, the authorship and provenance of Martinez' responses.
2. Location independent – the locations of Dr. Abbas, Martinez, and the referenced resources are irrelevant. Just as the location of a physical book does not change the content of the book, the contents of the verifiably identified requests and responses do not depend on where they happen to be created or stored.
3. Time independent – similarly, the validity of the search results and retrieved content does not depend on the time of retrieval because the messages and relevant contents are identified using content-based signatures, and the result of the cryptographic function used to calculate the content hash does not depend on the time of the calculation.

Note that commonly used communication methods (e.g., HTTP, FTP, email), can still be used to implement discovery and retrieval methods that use content signatures. For example, requests and

responses can be exchanged in HTTP messages, digital files, and electronic messages. Even analog communication methods (e.g., paper, spoken word) can be used, as long as a suitable, lossless encoding method is used to represent communicated content in a digital form and compute a content hash. Also note that Dr. Abbas and Martinez can represent human or machine actors.

Without content signatures, Dr. Abbas and Martinez have to trust, but cannot verify, the identities of the referenced (and retrieved) contents. Furthermore, without verifiable provenance, evaluating content's authenticity relies on subjective, often implicit assumptions of trust derived from, for example, reputation, personal experience, institutional affiliation, the domain name of the service, the delay between request and response, or the quality of the communication channel. Following our discussion of the World Wide Web earlier, we know that most, if not all of these assumptions cannot be easily verified using identifiers (e.g., URLs) that are prone to link rot and content drift, especially over long periods of time.

References

Ascher, J. S. & Pickering, J. Discover Life bee species guide and world checklist (Hymenoptera: Apoidea: Anthophila) (2020). Accessed at http://www.discoverlife.org/mp/20q?guide=Apoidea_species.

Elliott, M. J., Poelen, J. H. & Fortes, J. A. B. Toward reliable biodiversity dataset references. Ecol. Informatics 59, 101132, https://doi.org/10.1016/j.ecoinf.2020.101132 (2020).

Federhen, S. The NCBI Taxonomy Database. *Nucleic Acids Research* **40**, D134-D143, https://doi.org/10.1093/nar/gkr1178 (2011).

Poelen, J. H. & Elliott, M. J. Biodiversity dataset archive hash://sha256/8aacce08462b87a345d271081783bdd999663ef90099212c8831db399fc0831b (2021). Accessed at https://archive.org/details/biodiversity-dataset-archives.

Poelen, J. & Salim, J. A. globalbioticinteractions/nomer:, https://doi.org/10.5281/zenodo.6478468 (2022).

Poelen, J. H. Nomer Corpus of Taxonomic Resources hash://sha256/6224f259190590c7aed4784de2b27b3005eea0042ae02993eb591f7a0fe30d87137, https://doi.org/10.5281/zenodo.6473194 (2022).

Poelen, J. H., Elliott, M. J. & Alzuru, I. bio-guoda/preston: 0.5.4, https://doi.org/10.5281/zenodo.7651885 (2023).

Seltmann, K. *et al.* Announcing Big-Bee: An initiative to promote understanding of bees through image and trait digitization. Biodivers. Inf. Sci. Standards (2021). Accessed at https://escholarship.org/uc/item/0937b5gp.

Integrated Taxonomic Information System (ITIS) on-line database, https://doi.org/10.5066/F7KH0KBK (2004).
